# Liraglutide inhibits receptor for advanced glycation end products (RAGE)/reduced form of nicotinamide-adenine dinucleotide phosphate (NAPDH) signaling to ameliorate non-alcoholic fatty liver disease (NAFLD) in vivo and vitro

**DOI:** 10.1080/21655979.2022.2036902

**Published:** 2022-02-14

**Authors:** Jingquan Ji, Ming Feng, Yan Huang, Xiaohong Niu

**Affiliations:** a Department of Pathophysiology, Changzhi Medical College, Changzhi, Shanxi, China; b Department of Neurosurgery, Changzhi People’s Hospital, Changzhi, Shanxi, China; c Department of Biochemistry, Changzhi Medical College, Changzhi, Shanxi, China; d Department of Endocrinology, The Heji Affiliated Hospital of Changzhi Medical College, Changzhi, Shanxi, China

**Keywords:** Liraglutide, nonalcoholic fatty liver disease, RAGE, NOX2

## Abstract

The study was designed to investigate the effects of liraglutide and reveal its action mechanism associated with RAGE/NAPDH in NAFLD. The liver tissue was collected for HE, Masson, and ROS staining. Apoptosis levels were detected through TUNEL staining and ROS levels were evaluated through ROS staining. The expression levels of c-Jun N-terminal kinase (JNK) and transforming growth factor-β (TGF-β) were detected through Western blot. JNK and the expression of Collagenα1, Collagenα2 and connective tissue growth factor (CTGF) were detected through RT-qPCR and Western blot and the expression in mouse liver stellate cells (JS-1) cells were evaluated through immunofluorescence staining. We detected the effects of liraglutide on NAFLD in high-fat diet (HFD)-fed mice. Liraglutide treatment improved bridging fibrosis and liver function, as well as lessening ROS levels and the protein levels of RAGE, NOX1, NOX2 and NOX4. In PA and H2O2-induced AML12 cells, liraglutide treatment was able to decrease cell apoptosis, ROS levels and the levels of inflammatory factors including tumor necrosis factor (TNF)-α, interleukin (IL)-1β and IL-6, while it effects were reversed by the induction of RAGE overexpression or NOX2 overexpression. In JS-1 cells treated with medium culturing AML12 cells, liraglutide markedly suppressed cell proliferation and activation, while RAGE overexpression or NOX2 overexpression blunted these effects of liraglutide. Taken together, liraglutide exerts a protective role in improving liver injury caused by HFD, which could be related to decreased apoptosis and oxidative stress of liver cells, as well as decreased proliferation and activation of hepatic stellate cells through RAGE/NOX2.

## Graphical abstract

**Figure:**
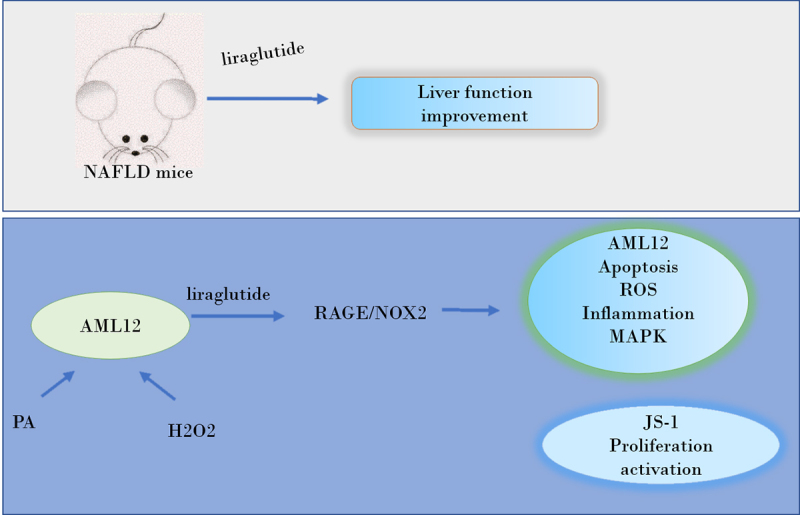

## Introduction

Nonalcoholic fatty liver disease (NAFLD) is characterized by excessive fat deposition in the liver and hepatic cell steatosis, and the pathogenesis excludes alcohol and other factors causing secondary hepatic steatosis [1,2]. There is no clinically effective treatment and lifestyle intervention is still the main treatment strategy for NAFLD. Drugs such as insulin sensitizers and antioxidants have certain effects on NAFLD, but their safety and side effects remain to be further evaluated [3,4]. Therefore, it is urgent to develop therapeutic drugs for NAFLD.

In NAFLD, due to inhibition of hormone-sensitive lipase activity, a large number of free fatty acids in adipose tissue were transferred to liver cells through systemic portal circulation. Excessive accumulation of toxic-free fatty acid promotes oxidative stress and increases the levels of lipid-free radicals such as reactive oxygen species (ROS) and reactive nitrogen in liver cells. Overproduction of reactive radicals leads to lipid peroxidation and induces oxidative stress, thereby inhibiting antioxidant mechanisms, and different pro-inflammatory cytokines such as TGF-beta, TNF-alfa, IL-8, Fas ligands and chemokines such as monocyte chemoattractant protein-1 are released [5]. Activation cascades of various pro-inflammatory and pro-fibrotic cytokines trigger the production of extracellular matrix by hepatic stellate cells [6]. Therefore, effective inhibition of oxidative stress and inflammation in liver cells is of great significance for inhibiting the activation of hepatic stellate cells and improving NAFLD.

Animal and human studies have shown that liraglutide promotes weight loss, improves insulin resistance, liver lipid deposition and liver steatosis [7]. Liraglutide can inhibit the expression of RAGE [8,9], which can be activated by processed food-derived advanced glycation end products to propagate the progression of NAFLD, leading to the accumulation of liver fat, resulting in inflammation, fibrosis, insulin resistance and other fatty liver complications [10,11]. Additionally, a study has shown that RAGE signals promote nonalcoholic fatty liver disease [12]. KEGG Pathway showed that RAGE could promote downstream NAPDH activity, thereby activating ROS and promoting the expression of inflammatory factors and TGF-β signals through MAPK family signaling. Meanwhile, NAPDH includes MOX1, NOX2, and NOX4 while NOX1 has been reported to be increased in NASH patients [13], and NOX2 is increased in nonalcoholic fatty liver mouse models [14]. Probiotics can inhibit NOX4 expression in NAFLD mice [15]. We predict that the effects of liraglutide against NAFLD is associated with RAGE/NAPDH signal. Therefore, this study was designed to determine whether liraglutide could inhibit hepatocyte injury by inhibiting RAGE/NAPDH signal, and reduce the activation of hepatic stellate cells, thereby elucidating the action mechanism of liraglutide against NAFLD.

## Method

### Animal experiment

C57BL/6 J mice (clean class), aged 4 weeks, purchased from Beijing Weitong Lihua Company (Beijing, China). The mice were fed under the conditions of relative humidity of 50% and at 23°C, in accordance with the national standards for experimental animals. Mice were grouped into three groups (each group: N = 8.) Mice were fed high-fat diet (HFD, 60% fat, 20% protein, Jiangsu Syony Pharmaceutical Biological Engineering Co. LTD, Nanjing, China) or a normal chow diet (Control) for 10 weeks. For HFD+Liraglutide group, the mice were injected with liraglutide for 2 weeks twice a day for 15 consecutive days [16]. After experiment, the body weight was recorded and fasting blood glucose was detected while Epididymal white fat was collected and weighed. The experiments were approved by the Ethics Committee of Changzhi Medical College.

### HE staining

The liver tissues were stained by HE staining. The sections were dewaxed in xylene I and II, respectively, for 15 min. The sections were dyed in hematoxylin for 30 seconds, depending on the specific situation. After being removed, the sections were washed with tap water for 15 minutes, and put into 1% hydrochloric acid alcohol for 10 seconds, then stained with eosin. The sections were observed under a light microscope.

### MASSON staining

The liver tissues were removed and fixed in 4% paraformaldehyde solution for 24 h, and then dehydrated and embedded with gradient ethanol and sectioned. The liver tissue sections of mice in each group were stained by Masson staining.

The detection of glutamic pyruvic transaminase (ALT) and glutamic oxaloacetic transaminase (AST)

The blood of mice was centrifuged at 4000 r·min^−1^ at 4°C for 5 min, and the supernatant was transferred to a new EP tube. The content of ALT and AST in blood was detected by automatic biochemical analyzer.

### ROS staining

Measurement principle DCFH-DA (2,7-dichlorofuorescin diacetate) is a fluorescent probe that can pass through freely. After entering the cell, it can be hydrolyzed by intracellular esterase to produce DCFH. Liver tissues were collected and stained using ROS kit and ROS levels in AML12 cells were evaluated through ROS kit following manufacture’s guidance (Elabscience).

### Western blot assay

The collected liver tissue and AML12 cells were lysed using RIPA Lysis buffer, then were centrifuged at 4°C at 14,000 r/min for 15 min. 30 μg protein sample was loaded and separated through SDS-PAGE electrophoresis. Then, proteins were transferred into PVDF membrane, which were then blocked with 5% skim milk powder, followed by incubation with primary antibodies (abcam, England) at 4°C overnight. Subsequently, the membrane was incubated with secondary antibodies (abcam, England) for 2 h at room temperature. The membrane was washed with PBST for 3 times, 10 min each, and the color was developed with hypersensitive ECL reagent (Millipore, USA). The grayscale of protein bands was analyzed by Image J software.

### Cell culture

AML12 cells or JS-1 cells were purchased from Procell (Wuhan, China) cultured in DMEM/F medium containing 10% FBS and 1% penicillin-streptomycin in 37°C with 5% CO2 constant temperature incubator. The medium was replaced every other day and the subculture and subsequent experimental operations were carried out when the cell growth density reached 80%~90%. AML12 cells were treated with 400 μM PA together with 200 μM or 400 μM H2O2 for 24 h.

### Real time quantitative PCR (RT-qPCR)

Trizol method was used to extract total RNA from each group of cells. The purity and concentration of total RNA were detected by microspectrophotometer (Thermo Fisher, USA), and the total RNA was reversely transcribed into cDNA. GAPDH was used as internal control and the relative expression of mRNA was calculated by 2^−ΔΔCt^ method.

### Cell counting kit-8 (CCK8) assay

AML12 cells were digested to prepare cell suspension and seeded into 96-well plates (100 μL/well) at a concentration of 2*10^4^ cells/well. Cell viability was measured by CCK-8 method according to manufacturer’s protocol (Beyotime, Shanghai, China).

### Plasmid transfection

AML12 cells were transfected with plasmids overexpressing RAGE or NOX2 using Lipo3000 according to manufacturer’s protocol (ThermoFisher). The plasmid information has been included in this manuscript. The sequences of the RAGE (NCBI Reference Sequence: NM_001271422.1.) or NOX2 (NCBI Reference Sequence: NM_007807.5) were cloned into pcDNA3.1. These plasmids were constructed and purchased from GenePharma (Shanghai, China).

### TUNEL staining

AML12 cells were collected for the detection of apoptosis levels through TUNEL staining (Beyotime, Nanjing, China) according to manufacturer’s protocol. Cells were fixed with 4% paraformaldehyde for 30 min at room temperature and then re-suspended with PBS containing 0.3% Triton X-100. After incubation at room temperature for 5 min, apoptotic cells were stained with TUNEL solution and nuclei were stained with DAPI. The cells were observed under a fluorescence microscope.

### Immunofluorescence assay

The JS-1 cells were fixed with 100% methanol (5 min), then permeabilized with 0.1% Triton X-100 for 5 minutes. Next, cells were blocked with 1% BSA/10% normal goat serum/0.3 M glycine in 0.1% PBS-Tween for 1 h. The cells were then incubated with primary antibody anti-α-SMA at 1/500 dilution (abcam, Englamd). Following incubation with secondary antibody, Nuclear DNA was stained with DAPI.

### Statistical analysis

The experimental data were analyzed for normal distribution and were shown in mean ± SD. For multi-comparisons in the in vivo and in vitro study, ANOVA analysis was performed among different groups, followed by Tukey's test. p < 0.05 was considered as statistical significance.

## Result

### Liraglutide suppresses the expression of RAGE/NOX in HFD mice

**Figure1:**
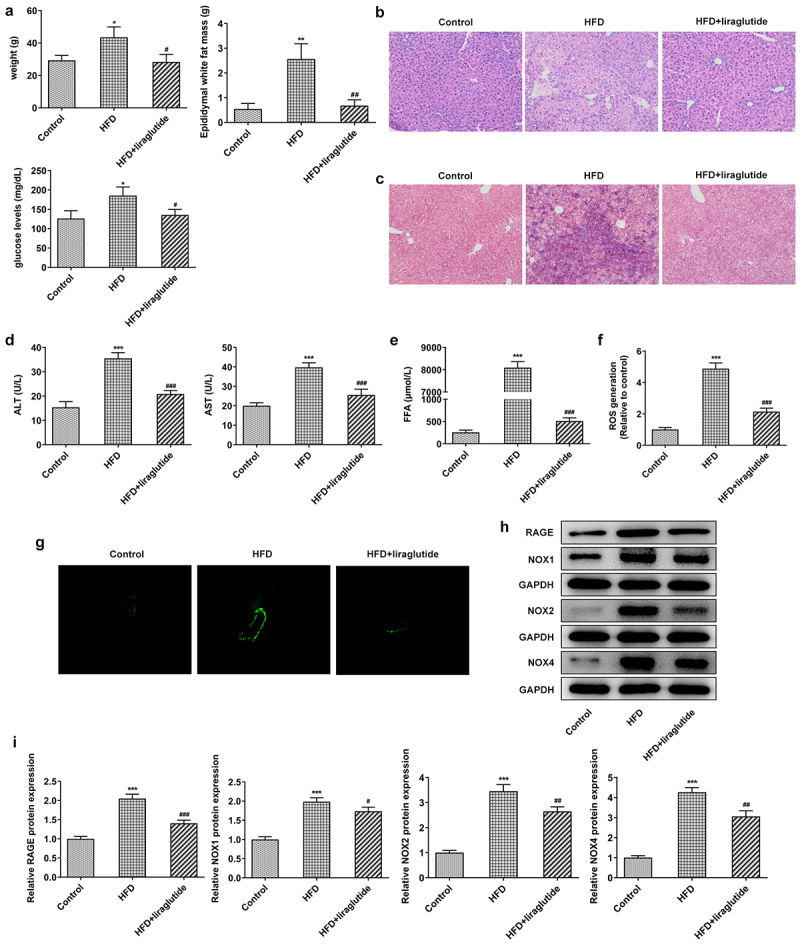
Liraglutide ameliorates liver injury in the HFD mice. (a) The weight, epididymal white fat mass and fasting blood-glucose levels. (b) HE staining of liver tissue. (c) Masson’s trichrome staining. (d) The levels of ALT and AST in the serum of mice. (e) FFA levels. (f, g) Representative images of ROS staining. (h) The levels of RAGE, NOX1, NOX2 and NOX4 in liver tissue of different group. The data were displayed as mean ± SD. n = 7 for each group. ***P < 0.001 compared with Control. ^#^ *P* < 0.05, ^##^ *P* < 0.01, ^###^ *P* < 0.001 compared with HFD group.

To determine the effect of liraglutide on RAGE/NOX, mice were fed with HFD or regular chow for 10 weeks and then administrated with liraglutide or saline. As shown in results, HFD diet increased weight, epididymal white fat mass and fasting blood-glucose levels compared with control group. Liraglutide administration contributed to significant decrease in weight, epididymal white fat mass and fasting blood-glucose levels when compared with HFD group (Figure 1(a)). The liver cells of normal control mice were polygonal, arranged regularly and closely, no obvious fat droplets were found in the liver cells, and the cells were in good condition. However, the liver cells of mice in the high-fat diet group were abnormal in shape, with different sizes and irregular arrangement (Figure 1(b)). After liraglutide treatment, the liver tissue pathology was significantly improved. HFD diet significantly increased bridging fibrosis and collagen, the levels of ALT and AST, as well as FFA levels. Liraglutide treatment reduced bridging fibrosis and collagen, the levels of ALT and AST, as well as FFA levels in the HFD mice (Figure 1(c–e)). ROS staining and Western blot for the protein levels of RAGE, NOX1, NOX2 and NOX4 were monitored in the liver tissue of mice. There were significant increases in ROS levels and the protein levels of RAGE, NOX1, NOX2 and NOX4 in the HFD mice compared with control group (Figure 1(f,h)). These levels were lower during liraglutide treatment in the HFD mice in comparison with control group. These results suggest the protective role of liraglutide in decreasing liver injury caused by HFD.

Liraglutide lessens RAGE/NOX2 expression in PA and H2O2-induced AML12 cells

**Figure 2.**
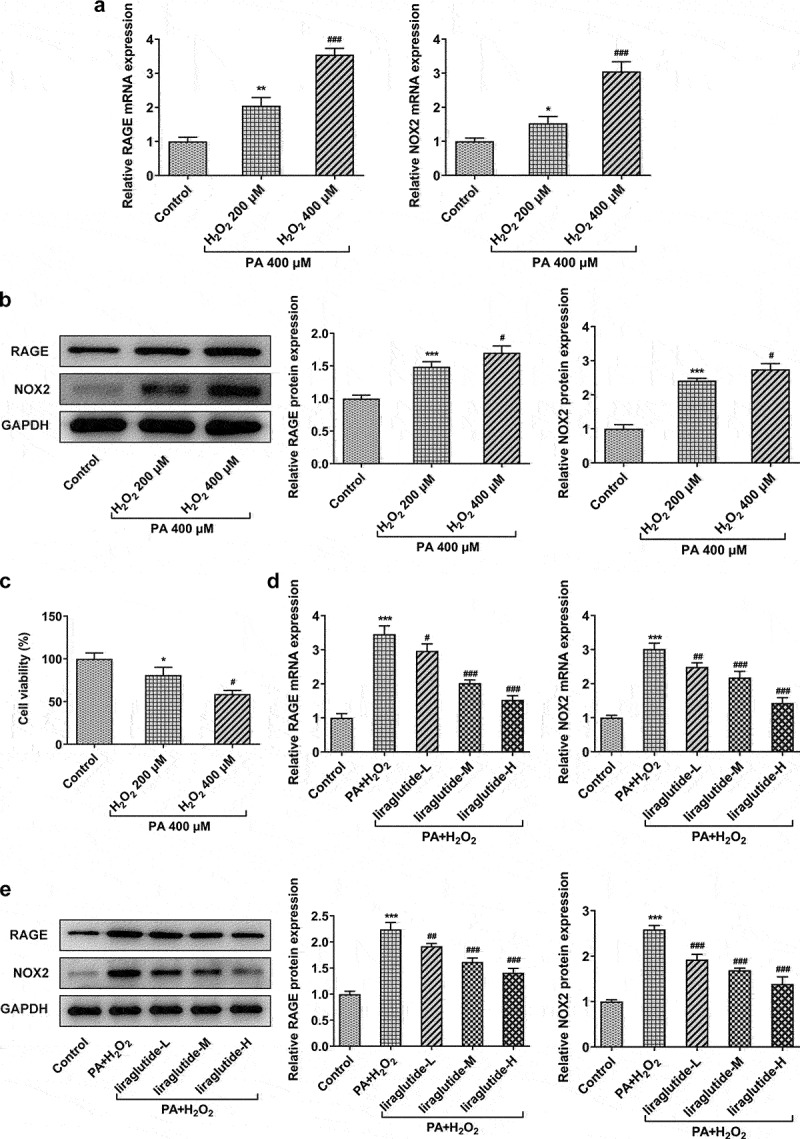
Liraglutide reduces the expression levels of RAGE and NOX2 in PA and H2O2-induced AML12 cells. (a) The mRNA levels of RAGE and NOX2. (b) The protein levels of RAGE and NOX2. (c) Cell viability through CCK8 assay. (d) The mRNA levels of RAGE and NOX2. (e) The protein levels of RAGE and NOX2. The data were displayed as mean ± SD. n = 7 for each group. *P < 0.05, **P < 0.01, ***P < 0.001 compared with Control. ^#^ *P* < 0.05, ^##^ *P* < 0.01, compared with H_2_O_2_ 200 µM group. ^###^ *P* < 0.001 compared with PA+ H_2_O_2_ group.

It can be seen from the above that liraglutide significantly inhibits the expression of RAGE/NOX2 signal. Next, we further determine the role of liraglutide in PA and H2O2-induced AML12 cells. The co-treatment of PA and H2O2 markedly increased the expression levels of RAGE and NOX2 in both mRNA and protein levels (Figure 2(a,b)). Decreased cell viability was observed in the co-treatment of PA and H2O2 compared to control group (Figure 2(c)). Furthermore, the co-treatment of PA and 400 μM H2O2 decreased the levels of RAGE and NOX2 levels and cell viability more obviously than that in 200 μM H2O2. Liraglutide treatment reversed the expression levels of RAGE and NOX2 in PA and H2O2-induced AML12 cells in concentration-dependent manner (Figure 2(d,e)).

### Liraglutide decreases apoptosis and ROS/JNK levels in PA and H2O2-induced AML12 cells

**Figure 3.**
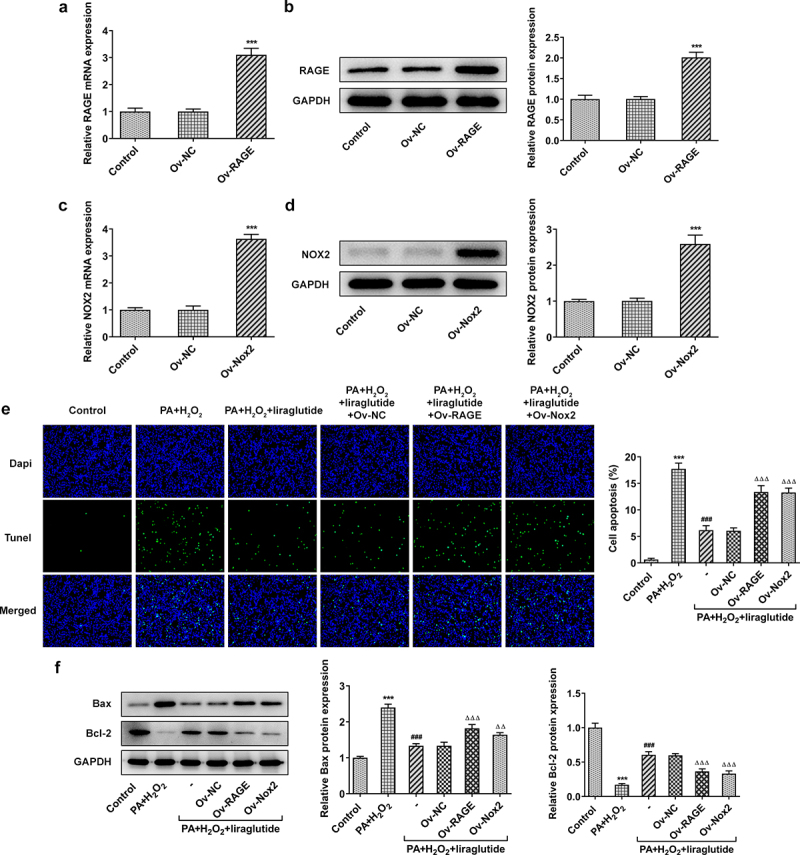
Liraglutide reduces apoptosis levels via RAGE/NOX2 in AML12 cells induced with PA and H2O2. (a, b) RAGE levels. (c, d) NOX2 levels. ***P < 0.001 compared with Ov-NC. (e) TUNEL staining for apoptotic cells. (f) Apoptosis-related protein levels. ***P < 0.001 compared with Control. ^###^ *P* < 0.001 compared with PA+ H_2_O_2_ group. ^ΔΔ^*P* < 0.001, ^ΔΔΔ^*P* < 0.001 compared with Ov-NC group.

**Figure 4.**
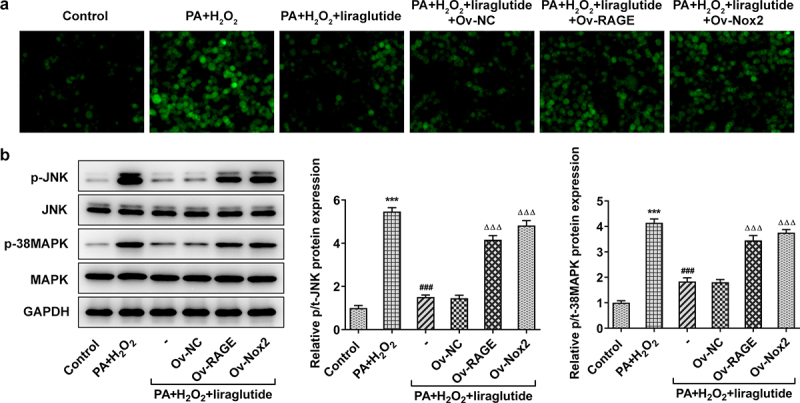
Liraglutide reduces ROS production and JNK signals via RAGE/NOX2 in AML12 cells induced with PA and H2O2. (a) ROS staining. (b) JNK and p38-MAPK expression levels. The data are represented as the mean ± SD. ***P < 0.001 compared with Control. ^###^ *P* < 0.001 compared with PA+ H_2_O_2_ group. ^ΔΔΔ^*P* < 0.001 compared with Ov-NC group.

In order to investigate the role of RAGE and NOX2 in the mechanism of liraglutide in PA and H2O2-induced AML12 cells, RAGE or NOX2 overexpression plasmids were constructed. Significant overexpression levels of RAGE or NOX2 were observed in Ov-RAGE or Ov-NOX2 transfected AML12 cells compared with control group (Figure 3(a–d)). Liraglutide treatment suppressed ML12 cell apoptosis caused by the co-treatment of PA and H2O2, but its effects were blunted when RAGE overexpression or NOX2 overexpression was induced (Figure 3(e)). Besides, liraglutide resulted in an increase in the expression of Bcl-2 and a decrease in the expression of Bax in AML12 cells challenged with PA and H2O2. But these effects were reversed by RAGE overexpression or NOX2 overexpression (Figure 3(f)). We further found that PA together with H2O2 promoted ROS production and increased the phosphorylated levels of JNK and p38-MAPK (Figure 4(a,b)). After liraglutide treatment, their phosphorylated levels were decreased in AML12 cells induced with PA and H2O2 (Figure 4(a,b)). However, these effects of liraglutide were blunted when the overexpression of RAGE or NOX2 was induced.

### Liraglutide inhibits PA and H2O2-induced inflammatory signal and the level of TGFβ protein through RAGE/NOX2

**Figure 5.**
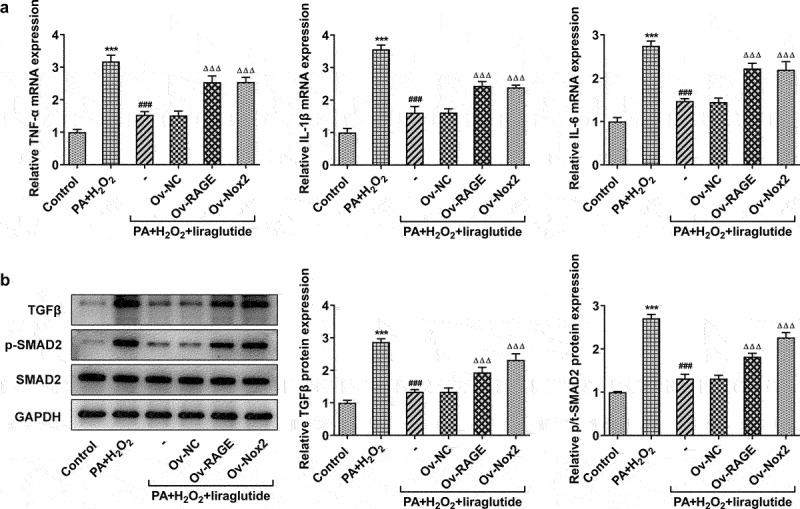
Effect of liraglutide treatment in inflammatory factor and TGFβ/SMAD. (a) The mRNA levels of TNF-α, IL-1β and IL-6. (b) The protein levels of TGF and SMAD2. Values are indicated as mean ± SD. ***P < 0.001 compared with Control. ^###^ *P* < 0.001 compared with PA+ H_2_O_2_ group. ^ΔΔΔ^*P* < 0.001 compared with Ov-NC group.

Obesity is a chronic inflammation, which could lead to liver injury [17]. To determine the impact and mechanism of liraglutide in inflammation on PA+H2O2-induced-AML12 cells, we performed RT-qPCR analysis and Western blot analysis. The results showed that the cotreatment of PA and H2O2 treatment had an effect on inflammation. The liraglutide treatment decreased an increase in the levels of TNF-α, IL-1β and IL-6 in the cotreatment group of PA and H2O2 (Figure 5(a)), while it led to a decrease in the protein levels of TGF and phosphate SMAD2 (Figure 5(b)). However, RAGE overexpression or NOX2 overexpression markedly reversed these effects of liraglutide.

### Liraglutide inhibits proliferation and activation of hepatic stellate cell

**Figure 6.**
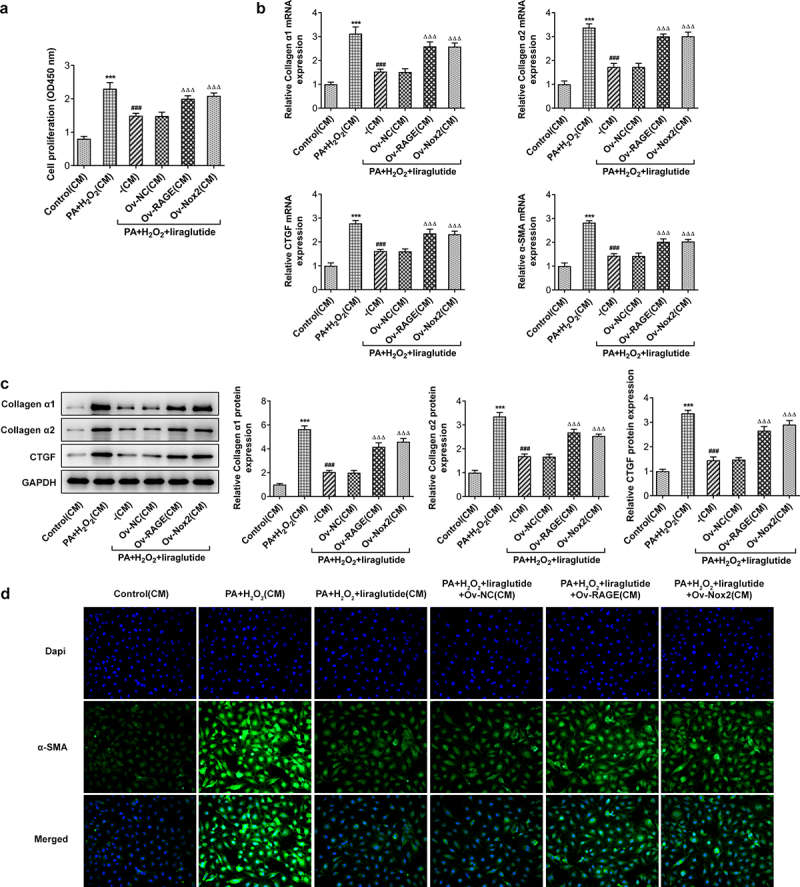
Effects of liraglutide in suppressing proliferation and the expression of Collagenα1, Collagenα2, CTGF and α-SMA of JS-1 cells via RAGE/NOX2. (a) CCK8 assay for the detection of cell proliferation. (b) Collagenα1, Collagenα2, CTGF and α-SMA expression through RT-qPCR. (c) Collagenα1, Collagenα2 C and TGF expression through Western blot assay. (d) Immunofluorescence staining for α-SMA. The data were indicated as mean ± SD. ***P < 0.001 compared with Control. ^###^ *P* < 0.001 compared with PA+ H_2_O_2_ group. ^ΔΔΔ^*P* < 0.001 compared with Ov-NC group.

To determine the effects of liraglutide on proliferation and activation of hepatic stellate cell, JS-1 cells were treated with medium of different group culturing AML12 cells indicated in Figure 5. As shown in the results of CCK-8 assay, the co-treatment of PA and H2O2 promoted JS-1 cell proliferation (Figure 6(a)). The cell proliferation rates were markedly decreased after treatment with liraglutide at high concentration, while the overexpression of RAGE or NOX2 was able to reverse this effect of liraglutide. Treatment with liraglutide led to decreases in the expression of Collagenα1, Collagenα2, CTGF and α-SMA in PA and H2O2-induced JS-1 cells, analyzed RT-qPCR, Western blot and Immunofluorescence staining (Figure 6(b–d)). Following the induction of the overexpression of RAGE or NOX2, cell proliferation and the Collagenα1, Collagenα2, CTGF and α-SMA expression were increased.

## Discussion

The pathogenesis of NAFLD has not yet been fully elucidated. Recent studies indicate that the occurrence and development of NAFLD is the result of a series of factors, such as hepatic cell lipid accumulation, insulin resistance and oxidative stress [18,19] [20]. Liver is the main site of fatty acid metabolism in the body. Excessive intake of high-fat food leads to energy metabolism imbalance and lipid metabolism disorder in liver [21,22]. A study found that liraglutide showed beneficial effects on improving lipid homeostasis and decreasing weight gain in HFD-fed mice depending on GLP1r expression in other organs except for liver [23]. There was a study also reporting that liraglutide modulates lipid metabolism by increasing cholesterol efflux associated with ABCA1 and ERK1/2 pathway. Furthermore, liraglutide modulates ERK1/2 pathway in HepG2 cells with HG challenge [24]. These suggested that liraglutide could exert a protective effect on the hepatocytes possibly by the other pathway independent on GLP-1 R. Our study shows that liraglutide lessens live injury in HFD-administrated mice, the mechanism of which is related to the decreased apoptosis and inflammation of liver cell and the activation of hepatic stellate cells via RAGE/NAPDH.

NADPH oxidase is a membrane-bound enzyme complex, and this family of proteins such as NOX1, NOX3, NOX4 and NOX5 is distributed in almost all organs, tissues and cells. Under abnormal conditions, such as the body being stimulated by cytokines, inflammatory mediators and growth factors in the environment, excessive expression of NOX would cause the production of a large number of ROS [25]. ROS participates in regulation of cell growth and apoptosis, which can regulate MAPK pathway including JNK and P38 to induce cell apoptosis [26–28]. The present data showed that liraglutide decrease apoptosis, ROS levels and the phosphate JNK and p38-MAPK while RAGE overexpression or NOX2 overexpression was able to blunt these effects, indicating that liraglutide modulated RAGE/NOX2 to reduce ROS levels, thereby suppressing apoptosis, which could be mediated through MAPK pathway. Apoptosis pathways including MAPK and nuclear factor-kappa B could be activated when oxidative stress results in the release of lipid peroxidation products in liver with excessive accumulation of lipid [29–31]. Apoptosis is involved in the progression of NAFLD. The co-treatment of PA and H_2_O_2_ was found to lead to lower cell viability and apoptosis induction in HepG2 cells [32], which is consistent with the present study in AML12 cells induced by PA and H2O2.

We found that JS-1 cell proliferation was decreased by liraglutide treatment via RAGE/NOX2 while the expression of Collagenα1, Collagenα2, CTGF and α-SMA was decreased. Activation of hepatic stellate cells is often the trigger of liver fibrosis [33,34]. The activated hepatic stellate cells induced synthesis of large amounts of cytokines and extracellular matrix [35–37]. In the current study, the improvement of liver fibrosis by liraglutide treatment could be partly due to the inhibition of hepatic stellate cells proliferation and activation.

## Conclusion

In summary, liraglutide has anti-NAFLD effects in vivo and vitro, which is achieved by modulating RAGE/NOX2, thereby reducing ROX, apoptosis and inflammation in liver cells and suppressing the proliferation and activation of hepatic stellate cells, which provides new sights in the mechanism of liraglutide in NAFLD.

## Highlights

1. Liraglutide improved liver fibrosis and function for NAFLD mice.

2. Liraglutide decreased apoptosis and ROS levels via RAGE/NOX2 in hepatocytes.

3. Liraglutide modulated MAPK signals via RAGE/NOX2 in hepatocytes.

4. RAGE/NOX2 modulation in hepatocytes by liraglutide suppressed activation of hepatic stellate cell.
